# Navigating Vaccine Misinformation: Assessing Newly Licensed Physicians’ Ability to Distinguish Facts from Fake News

**DOI:** 10.3390/epidemiologia6020027

**Published:** 2025-06-10

**Authors:** Elena Maria Ticozzi, Giovanni Gaetti, Luca Gambolò, Dario Bottignole, Pasquale Di Fronzo, Daniele Solla, Giuseppe Stirparo

**Affiliations:** SIMED—Società Italiana di Medicina e Divulgazione Scientifica, 43125 Parma, Italy

**Keywords:** vaccines, young physicians, medical education, preventive medicine

## Abstract

Background: Newly licensed physicians play a significant role in healthcare systems. However, they often lack knowledge about vaccine practices. This study aims to evaluate their ability to distinguish between fake news and the actual side effects of vaccines. Methods: We distributed a questionnaire to assess newly licensed physicians’ knowledge of side effects of vaccines and widespread fake news about them. We enrolled 317 newly licensed Italian physicians. Results: The average questionnaire score was 6.21/10 (SD = ±1.25). Work experience and age did not correlate with scores. Conclusions: Many participants were not able to distinguish between fake news and real vaccines’ side effects (e.g., the possible association between varicella vaccine and seizures). Furthermore, many physicians have been shown to believe in fake news. This lack of knowledge could lead to the inability to scientifically respond to anti-vaccinationists, thus increasing mistrust in medical counseling. Addressing knowledge gaps among recently licensed physicians is crucial to improving proper counseling and increasing public adherence to vaccination campaigns.

## 1. Introduction

The current structure of the Italian National Healthcare Service (SSN) allows physicians with a medical degree to practice and hold strategic roles, without the requirement of a specialization title [1]. This organizational model includes newly licensed physicians, defined as medical doctors within 12 months of obtaining their license, for whom theoretical and practical training still plays a pivotal role, as the experience and self-confidence of healthcare professionals are critical in managing different clinical scenario [2,3]. The importance of these young doctors within the SSN was highlighted during the COVID-19 pandemic, which negatively affected the SSN [4,5,6] and significantly disrupted medical training in medical schools all over the nation due to the adoption of restrictive measures for infection control [7,8]. Furthermore, during the peak of the pandemic period, young doctors were widely enrolled to assess COVID-19 outpatients [9]. Subsequently, many newly licensed doctors took part in the COVID-19 vaccination campaign, providing vital support to the Italian healthcare system during this time of crisis [10].

As a result, many universities have restructured their medical curricula to better prepare medical graduates for these specific topics, adopting innovative measures to effectively deliver medical training [11]. However, due to staff shortages, newly licensed doctors in Italy often start to work as freelancers, without receiving an adequate mentoring [1]. This situation highlights the critical importance of the knowledge and skills acquired during academic training, as they serve as a foundation for the immediate postgraduate practice period, easing the transition into the professional world. Therefore, it is essential to assess the specific knowledge of newly qualified doctors to design targeted training programs that enhance their competencies and expertise, ensuring that they are properly equipped to face professional challenges immediately after their graduation.

The Italian medical association SIMED (“Società Italiana di Medicina e Divulgazione Scientifica”, in Italian) is committed to medical and health education, with a particular focus on newly licensed physicians who perform various roles within the SSN. Besides their role during the COVID-19 pandemic, and due to the aforementioned staff shortage that the Italian healthcare system is experiencing, newly licensed physicians are often employed as substitutes for general practitioners, in Out-of-Hours Medical Care services, and in vaccination campaigns for the SSN.

However, research indicates that a significant number of newly licensed physicians and medical students have limited knowledge of vaccine practices [12,13]. As a result, they often struggle with independent decision-making and demonstrate low autonomy even in routine outpatient procedures, highlighting the need for enhanced training and educational initiatives in this critical area of healthcare. A lack of knowledge is often linked to increased vaccine hesitancy, even among medical students, which can have significant implications for public health and patient care [14]. Insufficient understanding of vaccine mechanisms, schedules, and safety profiles may lead to uncertainty in clinical decision-making, reluctance to address patient concerns effectively, and, in some cases, reduced confidence in recommending immunization strategies. This knowledge gap underscores the necessity of structured educational interventions to enhance vaccine literacy among future healthcare professionals [15].

Recognizing the importance of vaccine education, other researchers have implemented similar training models aimed at improving vaccine-related knowledge among medical students and young physicians. Preliminary findings from these interventions indicate promising improvements in self-confidence, clinical autonomy, and communication skills when discussing immunization with patients [15,16]. These results highlight the potential of structured educational programs to not only enhance medical knowledge but also equip future healthcare providers with the necessary skills to advocate for vaccines and counteract misinformation effectively.

Over the last few decades, the public has experienced an increase in the availability of technological resources that allow access to specific medical knowledge. While access to medical sources provides different benefits, it may also foster the circulation of false information, as experienced during the COVID-19 pandemic [17,18,19]. Since fake news may influence attitudes toward vaccination, leading to vaccine hesitancy, the ability to discern between misinformation and the real effects of vaccination is associated with a higher rate of vaccine acceptance [18,19,20,21]. Based on these premises, all healthcare workers play a significant role in promoting health education among the general population, as a multidimensional approach should ideally be adopted. Indeed, a comprehensive intervention should focus on direct patient education and advocacy, which are among the most impactful strategies for success [22,23].

As a foundation for any future initiatives, it is crucial to assess the current level of vaccine-related knowledge possessed by healthcare providers working in the frontline, including newly licensed physicians.

The aim of this study is to evaluate the ability of newly licensed physicians to differentiate between fake news and scientifically proven side effects of routinely administered vaccinations.

## 2. Materials and Methods

### 2.1. Study Design

This is a cross-sectional study conducted in two phases. The first phase was conducted in the spring of 2022, in which a qualitative assessment was performed on the main fake news circulating on search engines such as YouTube, Facebook, and Google. Additionally, a literature search and evaluation of Italian institutional portals were conducted to identify the real side effects of different vaccines. These results were discussed during a meeting with the Public Health working group of the “Società Italiana di Medicina e Divulgazione Scientifica” (SIMED). The heads of the working group created a survey of 20 questions, consisting of 10 fake news and 10 real effects. Public health professionals and experts in the field reviewed the questionnaire and selected five fake news and five real effects, which had a dichotomous answer format (yes/no). The selection of the 10 items was achieved through expert consensus during panel discussions. The final questionnaire consisted of statements on which the scientific community had already expressed an unequivocal opinion. 

In the second phase, the final questionnaire was distributed. Data were collected via an online questionnaire using Google Forms, which was distributed to all participants enrolled in a training course offered by our association. All participants had obtained a degree in medicine and surgery within the previous 12 months. Medical students and recent graduates from other courses were not eligible for inclusion. No demographic or geographical exclusion criteria were applied. The distribution took place during the first meeting of the course, which was not focused on vaccination. The course was open to all Italian newly graduated physicians and took place in the Italian city of Parma, in 2022. All attendees of the course were invited to compile the questionnaire on a voluntary basis after signing an informed consent. The questionnaire consisted of three demographic questions (gender, age, and work experience) and ten questions assessing theoretical knowledge. Each participant received a score from 1 to 10 based on the number of correct answers in the theoretical knowledge test. The anonymous questionnaires were then entered into a CSV (Comma Separated Value) file for analysis. A threshold of 60% of correct answers was selected as a passing score.

### 2.2. Statistical Analysis

Statistical analysis was conducted with R (Version 4.2). The significance level was considered with *p* < 0.05 for all statistical analyses. Categorical variables (gender and wrong answers) were presented as numbers and percentages, and continuous variables (total score, working experience, and age) as mean and standard deviation. Data were tested for normality using the Kolmogorov–Smirnov test. Categorical variables were analyzed using the Chi-square (χ^2^ test). Bivariate analysis was conducted using Spearman correlation to examine the relationship between the test score and demographic variables, including age and months of working experience. The z-test was conducted between the mean for continuous variables to assess the statistical significance of differences between groups. An odds ratio with 95% confidence interval was calculated to assess the likelihood of correctly answering questions related to vaccine-associated fake news compared to questions concerning real, evidence-based side effects or epidemiological data.

## 3. Results

### 3.1. Sample Characteristics

In 2022, a survey was conducted on 317 newly licensed physicians who were part of the SIMED network in Italy. The response rate was 100% (317 medical doctors). The survey was conducted online, and demographic information was collected.

The average age of the surveyed physicians was 26.93 years old (SD = ±3.27), and the average work experience was 2.44 months (SD = ±2.57). Most of the surveyed physicians (67.82%) were female. Table 1 reports age, mean score, percentage of physicians that passed the test (with a score equal or superior to 6), and the mean work experience in months divided by sex.

### 3.2. Questionnaire Result

The final questionnaire included 5 questions related to fake news (question 1, 5, 6, 8, and 10, as reported in Table 2) and 5 questions with real side effects and epidemiological data (question 2, 3, 4, 7, and 9 in Table 2).

The mean score of the surveyed physicians was 6.21 (SD = ±1.25). There was no significant difference in the scores between male and female physicians (6.22 vs. 6.18, OR=1.01, *p* = 0.94). Pearson correlation analysis revealed no significant correlation between scores and the physicians’ work experience (r = −0.108, *p* = 0.055), while a significant negative correlation was found for age (r = −0.13, *p* = 0.013). Table 2 reports the full questionnaire, with the number and percentage of physicians who answered each question correctly and incorrectly. Considering the typology of question, participants were more likely to answer correctly to questions related to fake news compared to questions about scientifically proven side effects or epidemiological data (OR 2.90, 95% CI 2.50–3.0).

## 4. Discussion

According to data from the Italian Medical Association (FNOMCEO), our study sample represented 3% of newly licensed physicians in 2022 [24]. The majority of our participants were women (n = 215, 67%), a proportion slightly higher than that observed in the overall population of Italian physicians (67% vs. 56%) [24].

Our analysis showed that sex did not influence the results. Age was negatively correlated with questionnaire scores, indicating that younger physicians tend to have better knowledge of vaccines. Although work experience was not significantly associated with differences in scores (*p* = 0.055), the *p*-value suggests a potential trend that warrants further investigation in future studies.

We observed considerable variability in the rate of incorrect responses across different questions. In three cases (questions 2, 5, and 7), more than half of the participants answered incorrectly, whereas in others (questions 1, 6, and 10), the error rate remained below 20%. These discrepancies suggest that while certain aspects of vaccination are well covered during medical education, others are inadequately addressed, leading to gaps in knowledge and uncertainty among students. This finding underscores the need for a targeted training program to help newly licensed physicians differentiate between vaccine misinformation and scientifically validated side effects. Such an initiative would be essential for enhancing their knowledge and ensuring accurate patient counseling.

Notably, 238 of the interviewed physicians (75%) were unaware that a hypotonic–hyporesponsive episode is a well-documented side effect of the diphtheria, tetanus, and acellular pertussis (DTPa) vaccine. This condition is characterized by a sudden reduction in muscle tone, a decreased responsiveness to stimuli and the presence of pallor, with an estimated incidence of 80 cases per 100,000 DTPa vaccinations [25,26]. Given its alarming presentation, it is crucial for physicians to recognize this reaction as vaccine-related and provide appropriate counseling to parents, ensuring they are informed and reassured.

A significant number of physicians did not recognize the association between the varicella vaccine and febrile seizures. Although this condition is generally benign, it can be alarming for patients and caregivers, therefore recognizing this potential side effect is essential for providing accurate counseling and appropriate management when needed, as the lack of recognition may impact the ability to handle these relatively common syndromes, leading to a loss of trust in healthcare professionals by patients and parents [27,28].

Approximately 60% of recently licensed physicians mistakenly believe that thimerosal, an inert substance used as a vaccine stabilizer, is harmful, a misconception largely propagated by anti-vaccine movements [29]. It is crucial to acknowledge that young physicians often work in roles that involve direct contact with vaccine-hesitant patients, such as in vaccination centers. Inadequate or incomplete mastery of specific topics may leave them unable to provide scientifically accurate responses, which could, in turn, weaken patients’ trust in medical counseling and compromise their willingness to be vaccinated.

Notably, we observed that participants were more likely to correctly recognize fake news compared to real side effects. Several factors may account for this finding, including a potential skeptical response from physicians toward alarmist narratives about vaccines, possibly shaped by the extensive spread of vaccine-related fake news over recent years. This attitude could result in the dismissal of patient concerns, compromising their relationship with the national healthcare system.

Vaccination training programs for newly qualified doctors could have significant organizational and public health implications, as physicians employed in hospitals play a crucial role not only in administering vaccines, but also in promoting vaccine awareness and confidence among patients. Their ability to provide accurate, evidence-based information can help counter misinformation and encourage higher vaccine uptake, especially among vulnerable populations. In addition, hospital-based physicians can serve as key advocates for influenza vaccination campaigns among healthcare professionals and promote a culture of vaccination within medical institutions. Increasing their knowledge in this area would enhance their ability to lead by example, ultimately contributing to improved vaccine coverage and patient confidence in immunization programs [30,31,32].

Simulation-based training is widely recognized as an important tool for increasing the autonomy of young doctors and is essential for improving their technical skills [33,34,35]. Integrating vaccination counseling simulations into medical education could be particularly valuable in strengthening physicians’ ability to communicate effectively with patients, address vaccine hesitancy, and counter argument misinformation. This type of hands-on training reinforces theoretical knowledge and builds confidence in dealing with real-life patient concerns, ultimately leading to improved counseling skills. In addition, despite the well-documented benefits of vaccines, adherence among healthcare professionals remains suboptimal, therefore strengthening their engagement through targeted education and simulation exercises could help address this issue and foster a culture in which physicians lead by example in vaccine advocacy [36,37,38].

Mass vaccination campaigns remain a cornerstone of public health strategy, ensuring widespread uptake of immunization against vaccine-preventable diseases. These campaigns play a crucial role in increasing vaccination coverage, protecting vulnerable populations, and preventing disease outbreaks, particularly during peak seasons of respiratory illnesses. Influenza-like illnesses (ILIs) have a profound impact on healthcare systems, not only due to their direct burden but also because their predictable seasonal surges strain healthcare resources, indirectly affecting the management and treatment of other diseases. Moreover, respiratory syndromes can directly influence the incidence and severity of other diseases by exacerbating underlying conditions, increasing the risk of complications, and contributing to poorer health outcomes in patients with chronic illnesses. This interplay highlights the critical need for proactive vaccination strategies to mitigate the overall disease burden and healthcare system sustainability [39,40,41,42,43].

Given their frontline role in these campaigns, equipping newly qualified doctors with both the knowledge and practical skills necessary for effective vaccine counseling is essential. Strengthening their ability to communicate the benefits of immunization can enhance public confidence, improve vaccine uptake among both the general population and healthcare workers, and ultimately reinforce the resilience of immunization programs and broader public health initiatives.

Several studies have assessed how education plays a pivotal role in shaping attitudes and influencing behavior regarding health, for both acute and chronic conditions [44,45,46,47]. Indeed, ensuring that patients recognize the value of clinical pathways requires not only comprehensive education but also effective counseling. By equipping both healthcare professionals and patients with the necessary knowledge and communication strategies, we can foster greater adherence to evidence-based care and enhance overall health outcomes.

Our study offers valuable insights, yet certain limitations must be acknowledged. First, the questionnaire was administered to physicians engaged in specialized postgraduate training, a cohort that may exhibit distinct educational gaps compared to the broader population of newly licensed physicians. While this focus provides a deeper understanding of specific learning needs, it may not fully represent the overall competency landscape of early-career doctors. Moreover, the absence of a test–retest analysis presents a notable limitation, as it precludes an evaluation of the tool’s reliability over time. Establishing the consistency of its measurements would enhance the robustness of our findings, ensuring that the observed trends are not merely incidental but truly reflective of underlying knowledge patterns. Finally, a comprehensive internal validity assessment was not performed, leaving room for further refinement in confirming the tool’s precision in measuring the intended construct. Future studies should address these aspects to strengthen the validity and applicability of our conclusions, ultimately paving the way for more targeted and effective educational interventions.

## 5. Conclusions

Despite the increasing importance of vaccination programs in public health and the widespread dissemination of misinformation, our results show that newly qualified doctors have a limited and inconsistent theoretical understanding of the subject. This knowledge gap is of particular concern given their role in patient education and vaccine advocacy. Notably, our analysis shows that knowledge levels do not vary significantly by gender, age, or years of professional experience, suggesting that these gaps persist across different demographic and professional backgrounds.

To effectively address vaccine hesitancy and improve vaccination rates, it is essential to implement targeted educational programs to strengthen the theoretical base of newly licensed physicians, focusing on providing comprehensive, evidence-based knowledge about vaccine safety, efficacy, and common misconceptions to equip physicians with the tools to confidently address patient concerns.

In addition to improving individual physician competence, these initiatives would have broader public health benefits by promoting a well-informed medical workforce capable of providing high-quality vaccine counseling. Strengthening physicians’ knowledge and communication skills would contribute to better-informed patients, increased vaccine uptake and, ultimately, higher vaccination coverage. Investment in such educational strategies is essential to ensure that physicians are adequately prepared to serve as trusted sources of vaccine information, to increase public confidence in immunization programs, and to reduce the impact of misinformation on vaccine uptake.

## Figures and Tables

**Table 1:** Demographic characteristics of the sample population.

Population N (%)	Mean Age (SD)	Mean Score (SD)	Passed (%)	Mean Work Experience in Months (SD)
Women 215 (67.82%)	26.84 (3.54)	6.22 (1.26)	151 (29.76%)	2.45 (2.54)
Men 102 (32.18%)	27.12 (2.63)	6.18 (1.23)	30 (29.41%)	2.43 (2.66)
Total 317 (100%)	26.93 (3.27)	6.21 (1.25)	94 (29.65%)	2.44 (2.57)

**Table 2:** Full questionnaire, with correct and wrong answers.

Question	Correct Answer N (%)	Wrong Answer N (%)
1. Has the association between the human papilloma virus (HPV) vaccine and fibromyalgia been demonstrated?	No 277 (87%)	Yes 40 (13%)
2. Has the relationship between diphtheria, tetanus, and acellular pertussis (DTPa) vaccine and hypotonic-hyporesponsive episode in children been demonstrated?	Yes 79 (25%)	No 238 (75%)
3. Has a relationship between the varicella vaccine and febrile seizures been demonstrated?	Yes 161 (51%)	No 156 (49%)
4. According to recent studies, the flu vaccination campaign adherence rate of healthcare workers is about 15–18%?	Yes 201 (63%)	No 116 (37%)
5. Are vaccines containing mercury (in the form of thimerosal) being withdrawn because they are unsafe?	No 126 (40%)	Yes 191 (60%)
6. Is there a relationship between the measles, mumps, and rubella (MPR) vaccine and sudden infant death syndrome?	No 303 (96%)	Yes 14 (4%)
7. Has the relationship between the measles vaccine and Encephalomyelitis been demonstrated?	Yes 119 (38%)	No 198 (62%)
8. Has the relationship between the flu vaccine and Guillain-Barré syndrome been demonstrated?	No 194 (61%)	Yes 123 (39%)
9. Has the relationship between vaccines and anaphylactic shock been demonstrated?	Yes 232 (73%)	No 85 (27%)
10. Has the relationship between the influenza vaccine and encephalomyelitis been demonstrated?	No 278 (88%)	Yes 39 (12%)

## Data Availability

Data are available upon reasonable request.

## Abbreviations

The following abbreviations are used in this manuscript:

SSN	Italian National Healthcare System
SIMED	Società Italiana Medicina e Divulgazione
FNOMCEO	Federazione Nazionale degli Ordini dei Medici Chirurghi e degli Odontoiatri
DTPa	Diphteria, Tetanus and acellular Pertusiss vaccine
